# Factors associated with SARS-CoV-2 vaccine hesitancy after stroke: a cross-sectional study

**DOI:** 10.1186/s12889-024-18922-y

**Published:** 2024-05-26

**Authors:** Peng Hu, Ying-Hai Zhu, Chuan-Chuan Bai, Wei Wang, Duo Li, Lei Cao, Yan-Qing Huang, Tian Heng, Xiao-Han Zhou, Tao Liu, Ya-Xi Luo, Xiu-Qing Yao

**Affiliations:** 1 Department of Rehabilitation, The Second Affiliated Hospital of Chongqing Medical University, Chongqing, China; 2 Chongqing Municipality Clinical Research Center for Geriatric Medicine, Chongqing, China; 3 Department of Rehabilitation, University-Town Hospital of Chongqing Medical University, Chongqing, China; 4 Cardiopulmonary Rehabilitation Centre, Taihe Hospital, Affiliated Hospital of Hubei University of Medicine, Shiyan, China; 5 Department of General Medicine, The Nanhua Affiliated Hospital, Hengyang Medical School, University of South China, Hengyang, China; 6 Department of Emergency Medicine, First Hospital of Yulin, Yulin, China; 7 Department of Rehabilitation, Guangzhou Rehabilitation Hospital of the Elderly, Guangzhou, China

**Keywords:** SARS-CoV-2, Vaccination status, Vaccine hesitancy, Stroke

## Abstract

**Background:**

The vaccination status of post-stroke patients, who are at high risk of severe outcomes from Severe Acute Respiratory Syndrome Coronavirus 2 (SARS-CoV-2), is a significant concern, yet it remains unclear. We aimed to explore the vaccination status, factors associated with vaccine hesitancy, and adverse effects after vaccination among post-stroke patients.

**Methods:**

This multi-center observational study enrolled hospitalized post-stroke patients from six Chinese hospitals (Oct 1, 2020 - Mar 31, 2021), examining vaccine uptake and self-reported reasons for vaccine hesitancy, utilizing logistic regression to investigate risk factors for vaccine hesitancy, and recording any adverse reactions post-vaccination.

**Results:**

Of the total 710 post-stroke patients included in the study, 430 (60.6%) had completed the recommended full-3 dose SARS-CoV-2 vaccination, with 176 (24.8%) remaining unvaccinated. The most common reasons for vaccine hesitancy were concerns about vaccine side effects (41.5%) and impaired mobility (33.9%). Logistic regression identified advanced age (aOR = 1.97, 95%CI: 1.36–2.85, *P* = 0.001), lower Barthel Index score (aOR = 0.88, 95%CI: 0.82–0.93, *P* = 0.018), higher Modified Rankin Scale score (aOR = 1.85, 95%CI: 1.32–2.56, *P* = 0.004), and poorer usual activity level of EuroQol 5-Dimension (aOR = 2.82, 95%CI: 1.51–5.28, *P* = 0.001) as independent risk factors for vaccine hesitancy. Approximately 14.8% reported minor adverse reactions, mainly pain at the injection site.

**Conclusion:**

We found that post-stroke patients have insufficient SARS-CoV-2 vaccination rates, with key risk factors for vaccine hesitancy including concerns about side effects, advanced age, and functional impairments. No severe adverse reactions were observed among the vaccinated population.

## Introduction

Severe acute respiratory syndrome coronavirus 2 (SARS-CoV-2) has unleashed a severe public health crisis worldwide, with 766 million cases and 6.9 million related deaths [1], devastatingly impacting people’s daily life, medical activities, and the economy [2–4]. The SARS-CoV-2 vaccine has proven effective in preventing SARS-CoV-2 infections, reducing the risk of reinfection, preventing the onset of severe pneumonia, and lowering severe illness rates and mortality [5–8]. It also helps establish herd immunity to curb the spread of epidemics [9, 10]. However, vaccinated individuals’ antibody levels gradually decrease over time; hence booster shots are necessary to maintain lasting immunity against virus variants [11, 12].

Individuals with chronic diseases such as cardiovascular disease, cerebrovascular disease, and chronic kidney disease are at a higher risk of severe illness and mortality after SARS-CoV-2 infection [13, 14]. Thus, it is crucial for these high-risk populations to receive SARS-CoV-2 vaccinations and boosters. Post-stroke patients (stroke survivors) are particularly susceptible to SARS-CoV-2 infection [15, 16]. They exhibit heightened levels of angiotensin-converting enzyme 2 (ACE2) in lungs, a critical receptor situated on lung epithelial cells, facilitating the initial binding and infection of SARS-CoV-2 [15, 16]. Furthermore, post-stroke patients undergo immune dysfunction and inflammatory activation, amplifying the risk of pulmonary infections, including susceptibility to SARS-CoV-2 infection [17]. Moreover, post-stroke patients face an increased susceptibility to severe SARS-CoV-2 outcomes, including pneumonia, hospitalization and death; They had a 2.5-fold increase in the rate of severe illness and a 1.12-fold increase in mortality after infection with SARS-CoV-2 [18–21]. Additionally, SARS-CoV-2 infection can lead to thrombotic complications [22], acting as an independent risk factor for ischemic stroke [23], possibly increasing the rate of stroke recurrence. SARS-CoV-2 binds to ACE2, which in turn blocks the neuroprotective effects of ACE2. These result in a vicious cycle between SARS-CoV-2 pneumonia and stroke [17]. Currently, there are approximately 12.2 million new cases of stroke annually worldwide, with 101 million existing post-stroke patients [24]. In China alone, about 3.94 million new stroke cases are reported each year, with a total of 28.76 million existing post-stroke patients [25]. Despite the significant number of post-stroke patients globally, data on their uptake and safety date of SARS-CoV-2 vaccines remains scarce. It is necessary to gain a better understanding of the situation regarding SARS-CoV-2 vaccination among these patients.

Vaccine hesitancy, defined as the delay or refusal of vaccination despite its availability, poses a substantial barrier to achieving high vaccination rates. It has been recognized as one of the top ten threats to global health [26, 27]. People with chronic neurological disorders, such as multiple sclerosis and other nervous system autoimmune disorders, exhibit particularly high levels of vaccine hesitancy towards SARS-CoV-2 vaccination [28]. Therefore, it is crucial to understand the factors associated with hesitancy among post-stroke patients to facilitate their active participation in vaccination programs. Moreover, monitoring adverse reactions post-vaccination in patients with chronic diseases is of significant importance. While studies have affirmed minimal risk of severe adverse events post-vaccination in patients with chronic conditions like coronary heart disease [29], the possible serious side effects in post-stroke patients following SARS-CoV-2 vaccination remain under-studied and unclear.

Based on the above considerations, we conducted a multicenter cross-sectional observational study aimed at exploring the SARS-CoV-2 vaccination status, adverse reactions, and factors associated with the hesitancy of the SARS-CoV-2 vaccine in stroke survivors. The findings of this study will provide a scientific basis for formulating vaccination strategies specifically for this population.

## Methods

### Study design and participants

This study is a multicenter cross-sectional study. Given the comprehensive rollout of China’s SARS-CoV-2 vaccination program starting from early April 2021, we opted to include patients who experienced a stroke before March 2021. This ensured that our participants did not contain individuals who were vaccinated before their stroke. Furthermore, to guarantee an ample sample size, we decided to set the time frame as the six months preceding March 2021, covering a broad range of stroke occurrences. Consequently, we recruited patients diagnosed with ischemic or hemorrhagic stroke during their hospitalization from October 1, 2020, to March 31, 2021. Patients were selected from six hospitals: the Second Affiliated Hospital of Chongqing Medical University (Chongqing Municipality), University-Town Hospital of Chongqing Medical University (Chongqing Municipality), Taihe Hospital (Hubei Province), The Nanhua Affiliated Hospital (Hunan Province), Guangzhou Rehabilitation Hospital of the Elderly (Guangdong Province) and the First Hospital of Yulin (Shanxi Province).

The inclusion criteria were as follows: (1) age between 20 and 80 years old; (2) diagnosis with ischemic or hemorrhagic stroke based on CT or MRI scans and medical history; and (3) voluntarily signed informed consent. Participants were excluded for any one of the following reasons: (1) comorbidity with HIV infection, autoimmune rheumatic diseases, hematologic diseases, cancer, which could affect vaccination; (2) current use of immunosuppressants; (3) concurrent consciousness disorders; and (4) unstable or critical illness.

This study was approved by the Ethics Committee of the Second Affiliated Hospital of Chongqing Medical University (NO: 2023 Science and Ethics Review No. 7), and was also registered on the Chinese Clinical Trial Registry prior to participants enrollment (NO: ChiCTR2300069529; 20/03/2023). All participants signed informed consent forms.

### Sample size and random sampling strategy

Based on Turner GM et al.’s research [30], the vaccination rate for SARS-CoV-2 among stroke survivors is 84.6%. For our study, we set *P* = 0.8, allowable error (δ) at 0.03 and α = 0.05. According to the formula n = (Z_1−α/2_/δ)²*P*(1 - *P*), we calculated a required sample size of 683. We aimed to enroll 820 participants, accounting for a 20% expected sample loss. Then, we adopted a stratified random sampling strategy to mitigate selection bias, taking into account the varying scales, grades, and number of stroke patient discharges across different hospitals. Specifically, we implemented a rigorous randomization process using a computer-generated random number table method to select approximately 110–160 patients per hospital within each stratum.

### Data collection

We conducted telephone surveys to collect data from participants, including demographic information such as gender, age, marital status, and educational background. We also collected data about stroke details including time of onset, type of stroke, and any occurrences of recurrence. Additionally, we recorded information of chronic comorbidities and medication use.

The SARS-CoV-2 vaccination status information was recorded, including the number of doses received and any adverse reactions experienced post-vaccination. For those who had not yet completed the recommended vaccination regimen, we recorded the reasons for incomplete vaccination.

To evaluate the current functional status of participants, we used several scales: The Modified Rankin Scale (mRS), Barthel Index (BI), and EuroQol 5-Dimension (EQ-5D). The mRS measures disability on a scale from 0 (no symptoms) to 6 (dead), with higher score indicating greater disability [31]. The BI assesses the ability to perform ten daily activities, including tasks such as eating, toileting, and mobility, with a total score ranging from 0 (total dependence) to 100 (total independence) [32]. The EQ-5D evaluates the health-related quality of life across five dimensions: mobility, self-care, usual activities, pain/discomfort, and anxiety/depression. Each dimension is rated on a three-point scale: 1 (no problems), 2 (some/moderate problems), and 3 (extreme problems) [33].

The vaccines currently in use in China are those authorized for emergency use as of January 15, 2022, which include inactivated vaccines (developed by Sinopharm Beijing, Sinopharm Wuhan, and Beijing Kexing Zhongwei), an adenovirus vector vaccine (from CanSino Biologics in Tianjin), and a recombinant SARS-CoV-2 vaccine (from Anhui Zhifei Longcom). These vaccines follow a regimen of two initial doses plus a booster shot [34]. Currently, the administration of the booster dose (third dose) has been widely implemented across China.

### Process of telephone survey administration

Each randomly selected participant was contacted by telephone between March 20, 2023, and April 20, 2023, by the principal investigator at each center, using the telephone number recorded in the electronic medical record system. If a participant could not be reached after five calls, they were excluded from the study.

Before initiating the telephone survey, the participant’s identity was confirmed, and it was clarified whether the participant would be responding personally or if a proxy would be involved. If the participant exhibited normal cognitive and verbal functioning, they completed the questionnaire themselves; otherwise, a caregiver acted as a proxy and completed the questionnaire. The interview content included the elements outlined in the “Data Collection” section. Participants who refusing to take part or those who were deceased were excluded from the survey.

### Outcomes

#### Primary outcome

We reported the status of SARS-CoV-2 vaccination uptake among post-stroke patients, which includes both the number of vaccinated individuals and the count of doses received as of April 20, 2023.

**Secondary outcomes**: These encompass two dimensions. Firstly, we identified factors that contributed to vaccine hesitancy, a complex phenomenon conceptualized from the perspectives of cognition or affect, behavior, and decision-making [35]. Adopting a behavioral perspective, vaccine hesitancy can span from individuals who accept all vaccines without hesitation to those who completely refuse them, including those who are unvaccinated or under-vaccinated [26, 35]. Reflecting this spectrum, we categorized our participants into two groups: the Vaccine Hesitant Group who had taken less than three doses of vaccination, and the Non-Hesitant Group who had completed the full three-dose vaccination. In the Vaccine Hesitant Group, we collected the self-reported reasons for incomplete vaccination and used logistic regression to investigate the factors associated with vaccine hesitancy.

Secondly, we recorded adverse reactions following vaccination, categorized into short-term adverse reactions (occurring within 14 days after vaccination) and long-term adverse reactions (developing 14 days or more after vaccination). Mild short-term adverse reactions include local reactions at the injection site (pain, redness, swelling, and enlarged lymph nodes) and systemic reactions (fever, dizziness, headache, fatigue, nausea, vomiting, diarrhea, rashes, changes in sleep patterns, mood changes, and appetite changes). Severe short-term adverse events include thromboembolism, myocardial infarction, anaphylactic shock, and stroke recurrence. Severe long-term adverse events consist of stroke recurrence and myocardial infarction.

### Statistical analysis

Quantitative data are presented as mean ± standard deviation (SD), while categorical data as frequency (percentage). To compare two groups with quantitative data, an independent sample *t*-test is used while a chi-square test or Fisher’s exact test is used for categorical data. The initial factors associated with vaccination are determined through univariate logistic regression analysis. Afterward, a multivariate logistic regression analysis is performed using the backward Likelihood Ratio (LR) method. Covariates included in the model comprise variables with a *P*-value < 0.05 in the univariate analysis. A two-sided *P*-value < 0.05 is considered statistically significant in all tests conducted. R 4.2.2 (http://www.r-project.org/) was utilized for all data processing and figure generation purposes.

## Results

### Demographic and clinical characteristics of the study population

**Fig. 1:**
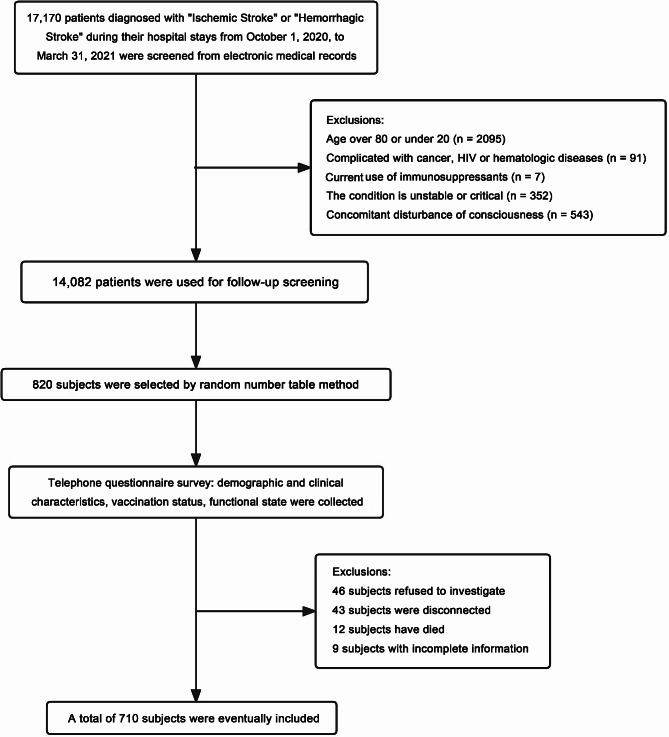
Flowchart of participants screen, randomization, and study progress

In total, we enrolled 820 participants previously diagnosed with “ischemic stroke” and “hemorrhagic stroke” and conducted telephone questionnaire surveys with patients or their proxies. Among them, 46 participants refused the survey, 43 were unreachable by phone, 12 were deceased, and 9 had incomplete information. Finally, 710 participants were included (Fig. 1). The average age of the participants was 61.0 ± 12.0 years, with 36.9% being female.

We compared the demographic and clinical characteristics of the Vaccine Hesitant Group and the Non-Hesitant Group (Table 1). We found that the Vaccine Hesitant Group was significantly older than the Non-Hesitant Group (62.8 vs. 59.8, *P* = 0.001). Moreover, a greater percentage of hemorrhagic stroke patients was found in the Vaccine Hesitant Group when compared to the Non-Hesitant Group (21.1% vs. 12.8%, *P* = 0.003). The residential locations of the two groups also showed marked differences (*P* < 0.001). Additionally, compared to the Non-Hesitant Group, the Vaccine Hesitant Group showed an increased prevalence of specific conditions including hyperlipidemia, diabetes, and chronic kidney disease (*P* < 0.05 for all). Medication use also differed significantly between the Vaccine Hesitant Group and the Non-Hesitant Group, with a higher proportion of aspirin (19.3% vs. 13.3%, *P* = 0.031) and insulin use (7.5% vs. 4.0%, *P* = 0.040) in the Vaccine Hesitant Group. Finally, the Vaccine Hesitant Group had a higher risk of stroke recurrence (15.4% vs. 9.8%, *P* = 0.025) and a higher rate of SARS-CoV-2 infection (96.8% vs. 93.5%, *P* = 0.048) compared to the Non-Hesitant Group.

**Table 1:** Demographic and clinical characteristics of the study population

Variables	Total (*N* = 710)	Non-Hesitant Group (*N* = 430)	Hesitant Group (*N* = 280)	*P*-value
Age, means (SD)	61.0 (12.0)	59.8 (11.4)	62.8 (12.6)	**0.001**
Gender, n (%)				0.790
Male	448 (63.1)	273 (63.5)	175 (62.5)	
Female	262 (36.9)	157 (36.5)	105 (37.5)	
Stroke subtypes, n (%)				**0.003**
Ischemic Stroke	596 (83.9)	375 (87.2)	221 (78.9)	
Hemorrhagic Stroke	114 (16.1)	55 (12.8)	59 (21.1)	
Marital status, n (%)				0.606
Married	660 (93.0)	398 (92.6)	262 (93.6)	
Non-married	50 (7.0)	32 (7.4)	18 (6.4)	
Education level, n (%)				0.243
Above middle school	154 (21.7)	87 (20.2)	67 (23.9)	
Middle school and below	556 (78.3)	343 (79.8)	213 (76.1)	
Residence, n (%)				0.525
Urban	531 (74.8)	318 (74.1)	213 (76.1)	
Rural	179 (25.2)	112 (26.0)	67 (23.9)	
Location, n (%)				**< 0.001**
Chongqing Municipality	289 (40.7)	182 (42.3)	107 (38.2)	
Hubei Province	139 (19.6)	120 (27.9)	19 (6.8)	
Hunan Province	96 (13.5)	14 (3.3)	82 (29.3)	
Guangdong Province	92 (13.0)	36 (8.4)	56 (20.0)	
Shanxi Province	94 (13.2)	78 (18.1)	16 (5.7)	
Comorbidities, n (%)				
Hypertension	430 (60.6)	269 (62.2)	161 (57.5)	0.178
Hyperlipemia	63 (8.9)	30 (7.0)	33 (11.8)	**0.028**
Diabetes	141 (19.9)	72 (16.7)	69 (24.6)	**0.010**
CAD	22 (3.1)	10 (2.3)	12 (4.3)	0.141
Chronic lung disease	22 (3.1)	10 (2.3)	12 (4.3)	0.141
Chronic kidney disease	9 (1.3)	2 (0.5)	7 (2.5)	**0.018**
DVT	9 (1.3)	4 (0.9)	5 (1.8)	0.319
Medications, n (%)				
Aspirin	111 (15.6)	57 (13.3)	54 (19.3)	**0.031**
Polivir/clopidogrel	40 (5.6)	25 (5.8)	15 (5.4)	0.796
Statins	158 (22.3)	89 (20.7)	69 (24.6)	0.217
Beta blockers	20 (2.8)	14 (3.3)	6 (2.1)	0.381
ACEI/ARB	154 (21.7)	95 (22.1)	59 (21.1)	0.747
CCB	158 (22.3)	102 (23.7)	56 (20.0)	0.244
Anticoagulant	33 (4.6)	18 (4.2)	15 (5.4)	0.469
Oral hypoglycemic agents	63 (8.9)	34 (7.9)	29 (10.4)	0.262
Insulin	38 (5.4)	17 (4.0)	21 (7.5)	**0.040**
Antiepileptic drug	26 (3.7)	16 (3.7)	10 (3.6)	0.917
Antispasticity medications	12 (1.7)	7 (1.6)	5 (1.8)	0.873
Diuretic	13 (1.8)	9 (2.1)	4 (1.4)	0.519
Stroke recurrence, n (%)	85 (12.0)	42 (9.8)	43 (15.4)	**0.025**
Experienced SARS-CoV-2, n (%)	673 (94.8)	402 (93.5)	271 (96.8)	**0.048**

CAD, coronary artery disease; DVT, deep vein thrombosis; ARB, angiotensin II receptor blocker; ACEI, angiotensin-converting enzyme inhibitor; CCB, calcium channel blocker.

### Vaccination status distribution

**Fig. 2:**
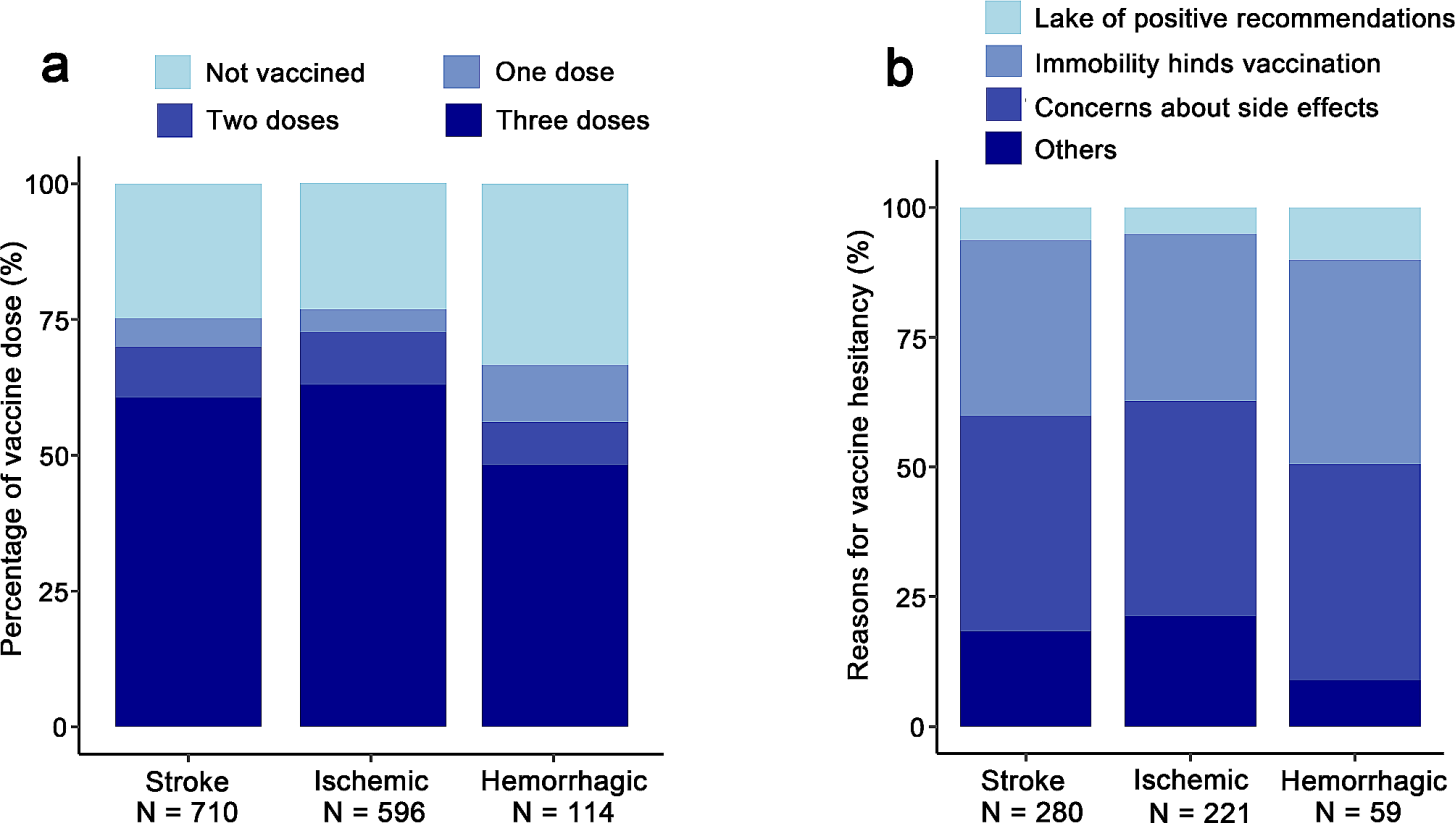
The SARS-CoV-2 vaccination rate and reasons for vaccine hesitancy among post-stroke patients. **(a)** the proportion of different vaccine doses received; **(b)** self-reported reasons for vaccine hesitancy from patients who have not completed the full 3-dose vaccination

Figure 2a shows the distribution of the number of vaccine doses received among the overall population of post-stroke patients and different subtypes. 430 patients (60.6%) had completed three doses of the SARS-CoV-2 vaccine; 67 (9.4%) had received two doses; 37 (5.2%) had received one dose; and there are still 176 (24.8%) who had not been vaccinated. Further analysis revealed a significant statistical discrepancy in the vaccination status between the ischemic stroke group and the hemorrhagic stroke group (*P* = 0.002). Specifically, a higher proportion of hemorrhagic stroke patients had not received the vaccine or had only received one dose compared to ischemic stroke patients (33.3% vs. 23.2%, 10.5% vs. 4.2%, respectively), and a smaller percentage had received three doses (48.2% vs. 62.9%). In addition, there were geographical variations in the percentage of patients who had received three doses of the vaccine. The rates at the central points of Chongqing, Hubei, Hunan, Guangdong, and Shanxi were 59.9%, 80.0%, 30.4%, 47.6%, and 66.7%, respectively.

### Self-reported reasons and objective factors associated with vaccine hesitancy

Further investigation into the reasons for vaccine hesitation among 280 participants in the Vaccine Hesitant Group revealed that the main reasons were concerns about vaccine side effects (41.5%), followed by impaired mobility affecting vaccination (33.9%). The lack of positive recommendations from medical staff accounted for 6.3% of the overall reasons. An additional 18.4% cited other reasons, including uncontrolled high blood pressure or poor physical health conditions making vaccination unsuitable (Fig. 2b). There were statistically significant differences in the reasons for vaccine hesitancy reported by ischemic stroke and hemorrhagic stroke patients. Hemorrhagic stroke patients reported more lack of active recommendation from medical staff (10.1% vs. 5.1%, *P* = 0.049), more impaired mobility affecting vaccination (39.3% vs. 32.2%, *P* = 0.022), but no statistical difference in concerns about vaccine side effects (41.4% vs. 41.6%, *P* = 0.27). Additionally, ischemic stroke patients reported more other reasons for not getting vaccinated (21.2% vs. 9.0%, *P* = 0.03).

We analyzed the objective risk factors for vaccine hesitancy (Table 2). Compared with the Non-Hesitant Group, the Vaccine Hesitant Group was older (OR = 1.73, 95%CI: 1.26–2.73, *P* = 0.001), more likely to have a hemorrhagic stroke (OR = 1.82, 95%CI: 1.22–2.72, *P* = 0.004), and have comorbidities including hyperlipidemia (OR = 1.78, 95%CI: 1.06–2.99, *P* = 0.029), diabetes (OR = 1.63, 95%CI: 1.12–2.36, *P* = 0.010), and chronic kidney disease (OR = 5.49, 95%CI: 1.13–26.6, *P* = 0.035). They were also more likely to be currently taking aspirin (OR = 1.56, 95%CI: 1.04–2.35, *P* = 0.031) and insulin (OR = 1.97, 95%CI: 1.02–3.80, *P* = 0.043). Furthermore, the Vaccine Hesitant Group had lower BI score (OR = 0.87, 95%CI: 0.84–0.91, *P* < 0.001), higher mRS score (OR = 1.35, 95%CI: 1.24–1.47, *P* < 0.001), worse EQ-5D levels (*P* < 0.05 for all), and a higher recurrence rate of stroke (OR = 1.68, 95%CI: 1.06–2.64, *P* = 0.026).

**Table 2:** Factors associated with vaccine hesitancy of SARS-CoV-2

Variables	Univariate analysis	*P*-value	Multivariate analysis	*P*-value
	Odds ratio (95% CI)		Odds ratio (95% CI)	
Age				
≤ 60 years	Reference			
> 60 years	1.73 (1.26–2.37)	**0.001**	1.97 (1.36–2.85)	**0.001**
Gender				
Male	Reference			
Female	1.04 (0.76–1.43)	0.790	1.00 (0.70–1.42)	0.961
Stroke subtypes				
Ischemic Stroke	Reference			
Hemorrhagic Stroke	1.82 (1.22–2.72)	**0.004**	1.51 (0.90–2.53)	0.116
Hyperlipemia	1.78 (1.06–2.99)	**0.029**	1.54 (0.86–2.76)	0.150
Diabetes	1.63 (1.12–2.36)	**0.010**	1.09 (0.67–1.78)	0.730
Chronic kidney disease	5.49 (1.13–26.6)	**0.035**	3.59 (0.67–19.33)	0.137
Aspirin	1.56 (1.04–2.35)	**0.031**	1.53 (0.98–2.37)	0.064
Insulin	1.97 (1.02–3.80)	**0.043**	1.21 (0.51–2.86)	0.661
Barthel Index score	0.87 (0.84–0.91)	**< 0.001**	0.88 (0.82–0.93)	**0.018**
Modified Rankin Scale score	1.35 (1.24–1.47)	**< 0.001**	1.85 (1.32–2.56)	**0.004**
EuroQol 5-Dimension				
Mobility	2.37 (1.82–3.08)	**< 0.001**	1.24 (0.67–2.27)	0.497
Self-care	2.50 (2.00–3.13)	**< 0.001**	1.30 (0.61–2.79)	0.498
Usual activities	1.94 (1.64–2.29)	**< 0.001**	2.82 (1.51–5.28)	**0.001**
Pain/discomfort	2.04 (1.36–3.08)	**0.001**	1.31 (0.79–2.18)	0.304
Anxiety/depression	1.63 (1.12–2.37)	**0.010**	1.02 (0.64–1.63)	0.925
Stroke recurrence	1.68 (1.06–2.64)	**0.026**	1.39 (0.84–2.28)	0.202

Then, we performed a multivariate logistic regression analysis and adjusted for potential confounding factors, including age, stroke subtype, comorbidities, BI score, mRS score, EQ-5D score, recurrence of stroke, current medication, and gender. We found that advanced age (adjusted OR = 1.97, 95%CI: 1.36–2.85, *P* = 0.001), higher mRS score (adjusted OR = 1.85, 95%CI: 1.32–2.56, *P* = 0.004), and worse usual activities level of EQ-5D (adjusted OR = 2.82, 95%CI: 1.51–5.28, *P* = 0.001) continued to be independent risk factors for vaccine hesitancy. In contrast, higher BI score (adjusted OR = 0.88, 95%CI: 0.82–0.93, *P* = 0.018) was a protective factor against vaccine hesitancy.

### Adverse reactions to vaccination

We investigated short-term adverse reactions in 534 participants who had received at least one dose of the vaccine. A majority of the participants (85.2%) reported no adverse reactions, while 14.8% reported experiencing at least one adverse reaction. In general, the adverse reactions were not severe, with the most common being pain at the injection site (9.0%), followed by changes in mood (3.6%), and redness or swelling at the injection site (1.7%). Other adverse reactions included fatigue (1.5%), fever (1.1%), rashes (0.7%), and changes in sleep patterns (0.7%). There were no statistically significant differences in adverse reactions between ischemic stroke and hemorrhagic stroke patients. No severe incidents of thromboembolism, myocardial infarction, stroke recurrence, or anaphylactic shock occurred within 14 days of vaccination in any of the participants (Table 3).

**Table 3:** Summary of the short-term adverse reactions that occurred after SARS-CoV-2 vaccination in post-stroke patients

Adverse events after vaccination, *N*(%)	Total (*N* = 534)	Ischemic Stroke (*N* = 458)	Hemorrhagic Stroke (*N* = 76)	*P*-value
None	455 (85.2)	393 (85.8)	62 (81.6)	0.336
Local adverse events				
Injection site pain	48 (9.0)	42 (9.2)	6 (7.9)	0.719
Local redness or swelling	9 (1.7)	8 (1.7)	1 (1.3)	0.999
Systematic adverse events				
Fever	6 (1.1)	5 (1.1)	1 (1.3)	0.999
Fatigue	8 (1.5)	6 (1.3)	2 (2.6)	0.318
Dizziness or headache	7 (1.3)	6 (1.3)	1 (1.3)	0.999
Appetite change	4 (0.7)	4 (0.9)	0 (0.0)	0.999
Sleep change	4 (0.7)	4 (0.9)	0 (0.0)	0.999
Mood change	19 (3.6)	14 (3.1)	5 (6.6)	0.170
Rashes	4 (0.7)	4 (0.9)	0 (0.0)	0.999

We also investigated the occurrence of serious adverse events among all participants from April 1, 2021, to April 20, 2023. In the Non-Hesitant Group, 42 participants (9.8%) experienced stroke recurrence, which was lower than in the Vaccine Hesitant Group (*n* = 43 or 15.4%, *P* = 0.025). There was no statistically significant difference in the incidence of myocardial infarction between the Non-Hesitant Group and the Vaccine Hesitant Group (2 cases vs. 3 cases, *P* = 0.388).

## Discussion

Our study demonstrates that despite the ongoing SARS-CoV-2 pandemic, nearly a quarter of post-stroke patients have remained unvaccinated against SARS-CoV-2, and over one-third have not completed the recommended three-dose SARS-CoV-2 vaccination regimen. The primary reasons reported for vaccine hesitancy include concerns about vaccine side effects and the impaired mobility affecting vaccination. Further analysis revealed that advanced age, lower BI score, higher mRS score, and poorer usual activity level of EQ-5D were independent risk factors for insufficient vaccination with less than three doses. Among vaccinated patients, 14.8% reported short-term adverse effects, mainly pain at the injection site, with no severe short-term side effects. Additionally, SARS-CoV-2 vaccination did not increase the incidence of long-term serious adverse events such as stroke recurrence and myocardial infarction.

Given that antibody levels decrease over time and new variants continue to emerge, booster shots are necessary to maintain immunity against SARS-CoV-2 and reduce mortality rates. Therefore it is crucial to promote continued SARS-CoV-2 vaccination efforts globally [11, 12]. Post-stroke patients, particularly those under 80 years old with ischemic or hemorrhagic stroke, have a higher infection rate and mortality after infection with SARS-CoV-2, and should be prioritized for vaccination [18–21]. Post-stroke patients have elevated levels of ACE2 in their lungs and altered systemic inflammatory responses, which facilitate SARS-CoV-2 binding and infection [15, 16]; and they often require continuous medical intervention, such as rehabilitation therapy, potentially making them more susceptible to nosocomial infection. Nevertheless, our research shows that 24.8% of post-stroke patients have not been vaccinated against SARS-CoV-2, surpassing the 15.4% reported by Turner GM et al. [30], which focused on the British population. They further noted that only 12% of individuals in their study received the full two vaccine doses, a rate lower than the 70.0% observed in our study [30]. The three-dose vaccination rate is 60.6%, lower than the general population in China (87%) [36]. The observed lower rate of SARS-CoV-2 vaccination in hemorrhagic strokes compared to ischemic strokes in our study may be attributed to greater functional impairment in hemorrhagic strokes. Research have indicated that hemorrhagic strokes generally exhibit greater severity compared to ischemic strokes, accompanied by a poorer functional recovery [37, 38]. This association was supported by logistic regression analysis followed, where hemorrhagic stroke emerged as a risk factor for vaccine hesitancy in univariate logistic regression analysis. However, the significance of the association between stroke subtypes and vaccine hesitancy disappeared after adjusting for BI score, mRS score, and EQ-5D score. In conclusion, for post-stroke patients, it is necessary to develop more specific healthcare strategies to increase the vaccination rate in this high-risk group and reduce their risk of infection and mortality.

Exploring factors that associated with SARS-CoV-2 vaccine hesitancy is crucial for developing targeted intervention measures to enhance vaccination rates. Factors associated with vaccine hesitancy in our study included concerns about vaccine side effects, functional impairment, and advanced age. Turner GM et al. reported that fear of vaccine side effects was a factor in vaccine hesitancy among post-stroke patients, aligning with one of our identified factors [30]. Notably, our results correspond to the widely recognized ‘3Cs’ model proposed by The World Health Organization Regional Office for Europe (WHO EURO) Vaccine Communications Working Group, which highlights three factors determining vaccine hesitancy: complacency, convenience and confidence [26]. In our study, patients who reported vaccine hesitancy cited fear of side effects as a major barrier, reflecting a lack of confidence in the vaccine’s safety. This suggests that strengthening education on vaccine safety for post-stroke patients and their families may alleviate these concerns and improve vaccination rates. We can implement targeted educational campaigns, distribute informative materials, and establish accessible communication channels to enhance vaccination communication and confidence, ultimately fostering increased vaccine acceptance among this population [39, 40]. Secondly, both self-reported reasons and objective reasons indicated that post-stroke functional impairment (measured by BI, mRS, and EQ-5D) or impaired mobility could be other significant reason for vaccine hesitancy among stroke survivors. This appears to be an issue of convenience in “3Cs” model, given that stroke patients often suffer from lasting disabilities, such as motor and language impairments, which can lower their quality of life, increase their dependence, and impact their ability or willingness to get vaccinated. A survey of 150,000 employees from a labor-intensive enterprise in Shenzhen, China also revealed that individuals with general or poor health status had a lower rate of SARS-CoV-2 vaccination [41]. Therefore, home-based vaccination services and mobile vaccination clinics may benefit post-stroke patients with severe functional impairment to improve vaccination rates [39, 40, 42]. In addition, advanced age was identified as another risk factor for vaccine hesitancy, a phenomenon that has been corroborated by other studies [43, 44].

After SARS-CoV-2 vaccination, some rare severe adverse reactions have been reported. These include thrombocytopenia, thromboembolism, myocardial infarction, and stroke [45, 46]. Although the incidence of these cases is low, they are still a concern for high-risk groups such as post-stroke patients. In our study, only 14.8% of post-stroke patients experienced mild and temporary adverse reactions to the SARS-CoV-2 vaccine, primarily pain at the injection site. There was no increase in rates of severe adverse events such as myocardial infarction or stroke recurrence after vaccination. These findings indicate that the SARS-CoV-2 vaccine has a favorable safety profile in post-stroke patients.

Our study is currently the largest multi-center observational study on SARS-CoV-2 vaccination among post-stroke patients, providing a detailed description of vaccination rates and safety, as well as a thorough investigation of factors associated with vaccine hesitancy. This provides valuable insights for increasing SARS-CoV-2 vaccination rates in post-stroke patients. However, this study has several limitations. First, although the sample size was carefully calculated, it remains relatively small given the heterogeneity of stroke patients. Second, as a cross-sectional study, it does not definitively establish a causal relationship between age, functional status, and vaccine hesitancy, and it lacks information on potential residual confounding factors associated with vaccine hesitancy, such as cognition about the vaccine and socioeconomic status. Future studies may also need to explore the causal relationship between age, functional status, and vaccine hesitancy, and to explore whether perceptions of vaccines, socioeconomic status, and other relevant aspects, are important factors influencing vaccine hesitancy. Third, our study predominantly involved a Chinese population. Given factors such as ethnicity, geography, policy, and vaccination types, it is essential to exercise caution in generalizing our findings to other countries and healthcare systems. Additional data from different ethnicities and regions are needed to strengthen the extrapolation of our findings. Lastly, selection of post-stroke patients from hospital electronic medical record systems could introduce selection bias.

## Conclusion

We discovered that nearly a quarter of post-stroke patients have remained unvaccinated against SARS-CoV-2, and over one-third are not fully vaccinated. Vaccine hesitancy among post-stroke patients may be attributed to subjective concerns about potential side effects, alongside objective factors such as advanced age and functional impairment. Due to the vaccination status and safety we observed in post-stroke patients, coupled with their vulnerability to SARS-CoV-2, it is reasonable to increase vaccination efforts in this population.

## Data Availability

The datasets used and/or analyzed during the current study are available from the corresponding author on reasonable request.
